# Spatiotemporal transcriptomic plasticity in barley roots: unravelling water deficit responses in distinct root zones

**DOI:** 10.1186/s12864-024-10002-0

**Published:** 2024-01-19

**Authors:** Alina Klaus, Caroline Marcon, Frank Hochholdinger

**Affiliations:** Institute for Crop Science and Resource Conservation, Crop Functional Genomics, University of Bonn, Friedrich-Ebert-Allee 144, 53113 Bonn, Germany

**Keywords:** Barley, Differential hub genes, Gene expression, RNA-seq, Root zones, Water deficit, WGCNA

## Abstract

**Background:**

Drought poses a major threat to agricultural production and thus food security. Understanding the processes shaping plant responses to water deficit is essential for global food safety. Though many studies examined the effect of water deficit on the whole-root level, the distinct functions of each root zone and their specific stress responses remain masked by this approach.

**Results:**

In this study, we investigated the effect of water deficit on root development of the spring barley (*Hordeum vulgare* L.) cultivar Morex and examined transcriptomic responses at the level of longitudinal root zones. Water deficit significantly reduced root growth rates after two days of treatment. RNA-sequencing revealed root zone and temporal gene expression changes depending on the duration of water deficit treatment. The majority of water deficit-regulated genes were unique for their respective root zone-by-treatment combination, though they were associated with commonly enriched gene ontology terms. Among these, we found terms associated with transport, detoxification, or cell wall formation affected by water deficit. Integration of weighted gene co-expression analyses identified differential hub genes, that highlighted the importance of modulating energy and protein metabolism and stress response.

**Conclusion:**

Our findings provide new insights into the highly dynamic and spatiotemporal response cascade triggered by water deficit and the underlying genetic regulations on the level of root zones in the barley cultivar Morex, providing potential targets to enhance plant resilience against environmental constraints. This study further emphasizes the importance of considering spatial and temporal resolution when examining stress responses.

**Supplementary Information:**

The online version contains supplementary material available at 10.1186/s12864-024-10002-0.

## Background

Barley (*Hordeum vulgare* L.) ranks fourth in global cereal production with 145.9 mio tons per year in 2021/22 [1]. It is used in various food products and beverages but mainly serves as fodder for livestock [2]. Barley is considered resilient against environmental constraints like salinity [3], or water deficit [4].

Nevertheless, global warming and thus prolonged drought periods [5] pose a major threat to global barley production [6]. Understanding the mechanisms underlying drought stress response and tolerance is essential to cope with these negative effects and will help to improve food security. Extensive research has been carried out to unravel the mechanisms of drought responses in plants and thus to increase crop tolerance [7–9]. Upon water deficit, plants initiate a multitude of molecular and physiological responses that aim to prevent detrimental effects caused by water loss. The phytohormone abscisic acid (ABA) was identified as a key player, orchestrating many regulatory processes, like stomatal aperture [10], including the regulation of gene expression via ABA-responsive element binding factors [11]. In contrast, dehydration-responsive element binding (DREB) proteins are part of the ABA-independent response complex but also act in gene regulatory processes [12]. The plasticity of gene expression enables the precise modulation of processes and pathways involved in stress response to water deficit and is the focus of many studies including crop species like maize [13], wheat [14], rice [15] and barley [16]. The study of transcriptomics facilitated by sequencing technologies, like RNA-sequencing [17], allows for studying drought responsiveness of all active genes of a tissue or organ. Moreover, advanced analytical tools, such as weighted gene co-expression network analysis (WGCNA, [18]), enabled the identification of stress-responsive gene groups by examining the expression patterns of genes. WGCNA is a systems biology method for describing correlations among large and quantitative data sets, such as RNA-seq. It can be used as an unsupervised analysis method to find modules of genes highly correlated in their expression pattern, which then can be associated with specific conditions or traits [18]. This correlation facilitates the network-based identification of candidate genes related to specific root zones and drought treatments. The genes, which are among the most highly connected ones within a module detected by WGCNA, are referred to as hub genes [18]. By integrating differential gene expression analysis and WGCNA, a comprehensive understanding of the complex molecular interactions underlying water deficit responses can be gained. Roots are the first plant organ to encounter water deficit. Therefore, they offer an ideal model to study early transcriptomic adaptations [19]. Roots can be separated into more specialized longitudinal root zones with distinct functions. While the root cap protects the root tip, the meristem harbors stem cells and thus provides new cells for growth. In the elongation zone, cells elongate, while in the differentiation zone, the most basal part of the root, cell differentiation takes place [20]. Though, it is well-established that each root zone exhibits distinct functions, many studies examine the effect of water deficit responses on the whole root level and thus, zone-specific mechanisms may be entirely masked [21].

In the present study, we focused on the effect of water deficit, simulated by polyethylene glycol (PEG8000), on root morphology and the root transcriptome of barley seedlings. High molecular weight organic osmotica such as PEG8000 (polyethylene glycol 8000), which cannot enter plant cells, can be utilized to mimic water deficit [22]. This allows generating defined water potentials to study plant responses under controlled water deficit conditions. Water deficit treatment of -0.8 MPa is in the mid-range of naturally occurring, plant-usable soil water potentials thus representing moderate drought stress [23]. We divided the root into three distinct longitudinal developmental zones: root cap and meristem, elongation zone and differentiation zone and performed RNA-sequencing after 6 h, 24 h and 48 h of water deficit. By this approach, we aim to provide a comprehensive overview of the spatiotemporal dynamics of gene expression patterns and fill the knowledge gap regarding zone-specific responses.

## Methods

### Plant material, growth conditions, and treatment

We pre-germinated seeds of the spring barley variety Morex for two days at 4 °C and then either transferred them to germination paper rolls [24] for RNA-sequencing or to germination paper-covered panels fitting into custom-built boxes [25] for phenotyping. We grew the plants in a climate chamber (Conviron, Winnipeg, Canada) at 20 °C at night (8 h) and 22 °C (16 h) at day and watered them with half-strength Hoagland solution [26]. After two days, we renewed the nutrient solution for control plants or exchanged it for a polyethylene glycol (PEG8000, Roth, Karlsruhe, Germany) solution with a water potential of -0.8 MPa to simulate moderate water deficit for stressed plants [27].

### Assessment of root growth under water deficit conditions

To ensure comparability, we selected only the three longest seminal roots per plant and measured the root length for seven consecutive days. We then calculated the average root length and growth rate of each plant at each time point and determined differences between control and water deficit plants by ANOVA in RStudio [28]. A mixed-effects model was used to analyze the data: lme(trait ~ treatment * time, random =  ~ 1 | id) with average root length or average root growth as trait responding to the interaction of the fixed-effect terms time (day 0 to day 7) and treatment (control and water deficit), correcting for a random-effect term id, that was used as an identifier for each plant. We performed *post-hoc* analyses with the emmeans package [29], which calculates the estimated marginal means (EMM) of the fitted model with treatment set as the specs argument and separated by time. Pairwise comparisons between EMMs of each treatment group were calculated and adjusted for multiplicity with the adjust = ”bonferroni” option. This way we handled every time point separately without neglecting the longitudinal character of the data. We used the package ggpubr [30] to visualize the data.

### RNA isolation and RNA-sequencing

We harvested root samples of seedlings grown in paper rolls for 6 h, 24 h and 48 h after water deficit stress induction. We then separated the roots into three distinct root zones: root cap and meristem, elongation zone and differentiation zone and immediately froze them in liquid nitrogen. The boundaries between the meristematic zone and the elongation zone were estimated based on previously analyzed longitudinal sections by Kirschner et al. [31], where the transition started around 1 mm from the root tip, while the boundaries towards the differentiation zone were marked by the first appearance of root hairs. In total, we obtained 54 samples with three biological replicates for all treatment-by-root zone-by -time point combinations with a pool of 30 roots for each biological replicate. We extracted total RNA with the RNeasy Mini Kit (Qiagen, Hilden, Germany) according to the manufacturer’s instructions and assessed RNA quality and integrity with a NanoDrop (Thermo Fisher Scientific, Waltham, MA, USA) and a BioAnalyzer (Agilent RNA 6000 Nano Chip, Agilent Technologies, Santa Clara, CA, USA). All samples exceeded a RNA integrity number value of 8.1. The RNA samples were sequenced on a NovaSeq 6000 sequencing platform (Novogene, Cambridge, UK) using a paired-end 150 bp strategy.

### Processing of raw sequencing data

We performed quality trimming of raw reads obtained from Novogene with trimmomatic v0.39 [32]. Trimmomatic was run in paired-end mode with the following options: ILLUMINACLIP:adapter.fa:2:30:10 LEADING:3 TRAILING:3 SLIDINGWINDOW:4:15 MINLEN:60. Reads > 60 bp were retained for subsequent processing. We then quantified transcript abundances with the pseudo alignment tool kallisto v0.46.0 [33] using the kallisto quant command with default options. The index was built from the transcriptome of Morex v3 (Hv_Morex.pgsb.Jul2020.HC.cds.fa; https://doi.ipk-gatersleben.de/DOI/b2f47dfb-47ff-4114-89ae-bad8dcc515a1/21172880-2956-4cbb-ab2c-5c00bceb08a2/0). We used the tximport package [34] to import transcript abundance quantification files from kallisto to R Studio including the option countsFromAbundance = ”lengthScaledTPM” to account for gene length and sequencing depth biases between samples. The resulting counts were then used for subsequent analyses. Kallisto uses expectation maximization instead of aligning reads to the reference, thus, reads that would be omitted due to multi-mapping in other approaches are now equally distributed between compatible transcripts. Hence, we filtered out gene models showing this equal distribution of counts across samples in R studio [28] before downstream analyses. The raw sequencing data were deposited in the NCBI SRA under BioProject accession number PRJNA988922.

### Analysis of differentially expressed genes

We analyzed the obtained read counts as previously described in Osthoff et al. [25]. In brief, we filtered read counts to only include active genes, i.e. genes with more than 0.4 counts per million reads in at least 3 samples. Then, we defined a linear model including a fixed effect for the combined factor treatment, time and root zone and transformed it with voom [35]. For visual representation of the sample relationships by spatial arrangement, we used a multi-dimensional scaling plot. We employed the R package limma [36] to fit the linear model and shrink the standard errors towards a common value with an empirical Bayes approach [37]. Contrasts between control and water deficit samples were always drawn from the same time point and root zone to mainly focus on the treatment effect. We adjusted the false discovery rate (FDR) to < 5%. Only gene models that displayed a |log_2_ FC|≥ 1 and FDR < 5%, were considered significantly differentially expressed.

### Weighted gene co-expression network analysis

We used a weighted gene co-expression network analysis [18] to find clusters of highly connected genes within the dataset derived from RNA sequencing of the different root zones. To set the focus on the treatment response, we carried out the co-expression analysis separately for each root zone. We filtered each count matrix for active genes by a cut-off ≥ 50 reads per gene model and used the function pickSoftThreshold with networkType = ”unsigned” to pick the best-fitting power value for calculating the adjacency matrix. The selected powers were eight for the meristematic, seven for the elongation and 12 for the differentiation zone matrix. We manually constructed the networks by first calculating an adjacency matrix. Then, we calculated the topological overlap matrix using the TOMsimilarity command on the adjacency matrix. We subtracted the values of the topological overlap matrix from one to calculate the dissimilarity matrix. To generate a clustered gene tree based on the dissimilarity matrix we used the command flashClust. We set the minClusterSize to 30 to avoid small clusters and used a dynamic approach to form clusters of branches that are highly similar with cutreeDynamic and the deepSplit = 2 option. We converted cluster allocations to a color scale and used this color scale to calculate the module Eigengenes. Module Eigengenes represent the overall expression patterns of genes within their modules. We calculated the dissimilarity of these eigengenes and clustered them with flashClust. Then, we merged close modules with cutHeight set to 0.3 for all root zones. For visualization of the obtained hierarchical clustering, we employed the plotDendroAndColors function. To identify modules that are correlated to the water deficit treatment, we computed the Pearson correlation coefficient between module eigengenes and traits (treatment-root zone-combinations). Since the examined trait data was qualitative and not quantitative, we used a presence-absence matrix in the correlation analyses.

We used the module membership or intramodular connectivity, which is the correlation between an individual gene and the respective module eigengene and the gene significance, which is the correlation between the expression of an individual gene and the trait, to identify hub genes within modules that showed a significant correlation with water deficit treatments. High gene significances indicate a higher biological relevance of the gene regarding the trait of interest, while high module membership indicates that a gene is highly connected to other genes within a selected module [18]. All genes that showed a module membership and a gene significance > 0.8 were considered hub genes. We then compared these hub genes to the sets of differentially expressed genes to identify differential hub genes that are consistently associated with water deficit across different analysis methods. To determine whether the observed overlap deviates from the expected overlap, we used either Fisher’s exact test (*n* < 5) or a chi-square test (*n* ≥ 5).

### Gene Ontology (GO) enrichment analysis

To further decipher the function of identified differentially expressed genes and differential hub genes, we used the Gene Ontology knowledgebase [38, 39]. We assigned ontology terms to the differentially expressed genes and carried out an enrichment analysis using the topGO package [40] in R studio. The degree of enrichment was determined with Fisher’s exact test. We used the algorithm = “weight01” option to reduce the number of false positives without missing too many true positives. Then, we filtered the obtained lists of enriched terms with REVIGO [41] to remove redundant terms with default settings. For visualization of significantly enriched gene ontology terms identified in the differential expression analyses, we used ggplot2 [42]. For visualization of enriched ontology terms identified in the co-expression analyses, we used Cytoscape [43]. To ensure accessibility, colors were chosen according to the viridis scale [44].

## Results

### Water deficit leads to decreased growth rates resulting in root length reduction

**Fig. 1:**
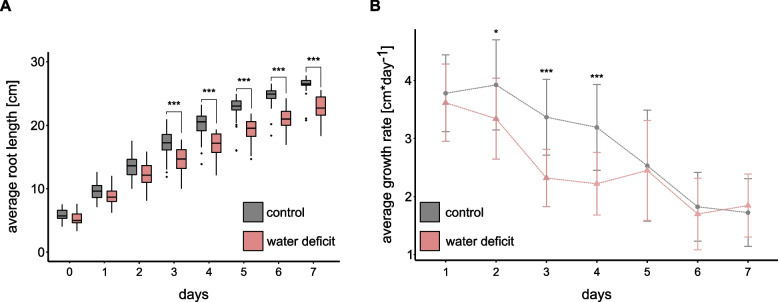
Effect of water deficit on barley seedling root traits. Values are averaged over the three longest roots for each sample. Grey color represents control, red color water deficit samples with *n* = 25 plants each. Differences between control and water deficit samples were determined by ANOVA using a mixed linear model that included the random effect term id used as a plant identifier (lme(trait ~ treatment*time, random =  ~ 1|id). Post hoc analyses were carried out based on estimated marginal means for each time point separately in pairwise comparisons between control and water deficit samples. **A** Comparison of root length. **B** Comparison of root growth rates. Bars represent the standard deviation of the means. *: *p* < 0.05; **: *p* < 0.01, ***: *p* < 0.001

To study the response of seminal root growth to water deficit, we monitored the average root length and growth rate of barley seedlings under control and moderate water deficit conditions (PEG8000, -0.8 MPa) for seven days and calculated significant pairwise contrasts between treatment groups based on their estimated marginal means (EMMs; Fig. 1). Between three and seven days of treatment, the average root length of water-deficit plants was between 15 to 20% shorter compared to the control group (Fig. 1A). For average root growth, we observed significant differences already after two days of treatment. From day five until the end of the experiment on day seven, both groups exhibited similar growth rates (Fig. 1B).

### Developmental and zone-dependent plasticity of the seminal root transcriptome upon water deficit

**Fig. 2:**
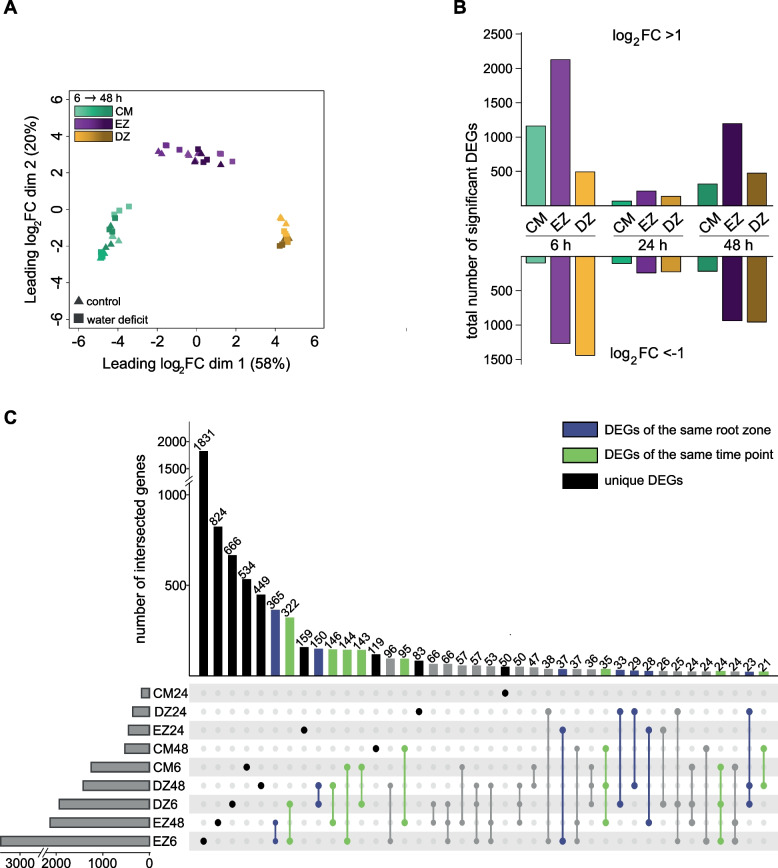
Sample relationship and differential gene expression. **A** Multidimensional scaling plot of seminal root tissue transcriptomes. **B** Number of differentially expressed genes (DEGs) identified in each root zone-by-time point combination. Bars represent up-regulated (log_2_FC > 1) and down-regulated (log_2_FC < -1) DEGs with an FDR < 5%. **C** Comparison of DEGs across root zones and time points. Intersections are marked by connecting lines between samples. The total number of intersected genes is indicated above each bar. DEGs that are unique to their root zone-by-time combination are marked in black. Intersections of DEGs from the same root zone across different time points are marked in blue, intersections of DEGs from the same time point across different root zones are marked in green, all other intersections are marked in grey. CM: root cap and meristem; EZ: elongation zone; DZ: differentiation zone

We surveyed the transcriptomic dynamics of barley seminal root zones subjected to water deficit (-0.8 MPa) and to control conditions for 6 h, 24 h and 48 h (see methods), hence before phenotypic changes in root length between the treatments were manifested (compare with Fig. 1A, 0 – 2 days). Changes in root growth rates were already visible after 48 h of treatment (Fig. 1B, day 2) and adaptations that underlie these changes in growth rate are thus included in the transcriptomic analysis of time point 48 h. Subsequently, we sampled three different longitudinal zones of seminal roots: the root cap and meristem, the elongation zone and the differentiation zone for all treatment-by-time point combinations in three biological replicates. For RNA-sequencing, we isolated RNA from these samples and converted them into cDNA libraries for sequencing (see methods). We then pseudo-aligned the obtained reads to the reference genome annotation of the barley cultivar Morex (v3) with an average overall rate of 87%. Details regarding individual pseudo-alignment rates and quality-based removals are summarized in Table S1. After removing duplicated, lowly expressed and inactive gene models (see methods), each library retained > 20 million reads for further analyses (Figure S1). We explored the transcriptomic kinship relation between samples in a multidimensional scaling (MDS) plot (Fig. 2A). The spatial arrangement of samples on the x-axis mirrored the distribution of root zones along the root axis from the root tip to the differentiation zone and explained 58% of the overall variance. Replicated samples from each root zone clustered together under control and water deficit conditions. Hence, differences between seminal root zones were more distinct than those between treatments. To identify genes differentially regulated in response to water deficit treatment, we computed pairwise contrasts between treated and control samples for each root zone-by-time point combination. The total number of differentially expressed genes (|log_2_ FC|> 1 and FDR < 5%) varied widely between root zones and time points (Fig. 2B, Figure S2). The highest number of water deficit-responsive genes (6580) was identified after 6 h of treatment. After 24 h of treatment, the number of water-deficit-responsive genes declined to 983 and increased again to 4091 differentially expressed genes after 48 h. When comparing sets of genes differentially expressed between control and water deficit conditions, large proportions of these genes were unique for their respective zone-by-treatment duration combination (Fig. 2C, highlighted in black). We observed a substantial overlap of genes differentially expressed between control and water deficit conditions at the same time point in different root zones (Fig. 2C, highlighted in green). Similarly, we identified genes, that were differentially expressed between control and water deficit conditions after different treatment durations in only one root zone (Fig. 2C, highlighted in blue).

### GO enrichment analysis highlights the complex and dynamic nature of water deficit responses

**Fig. 3:**
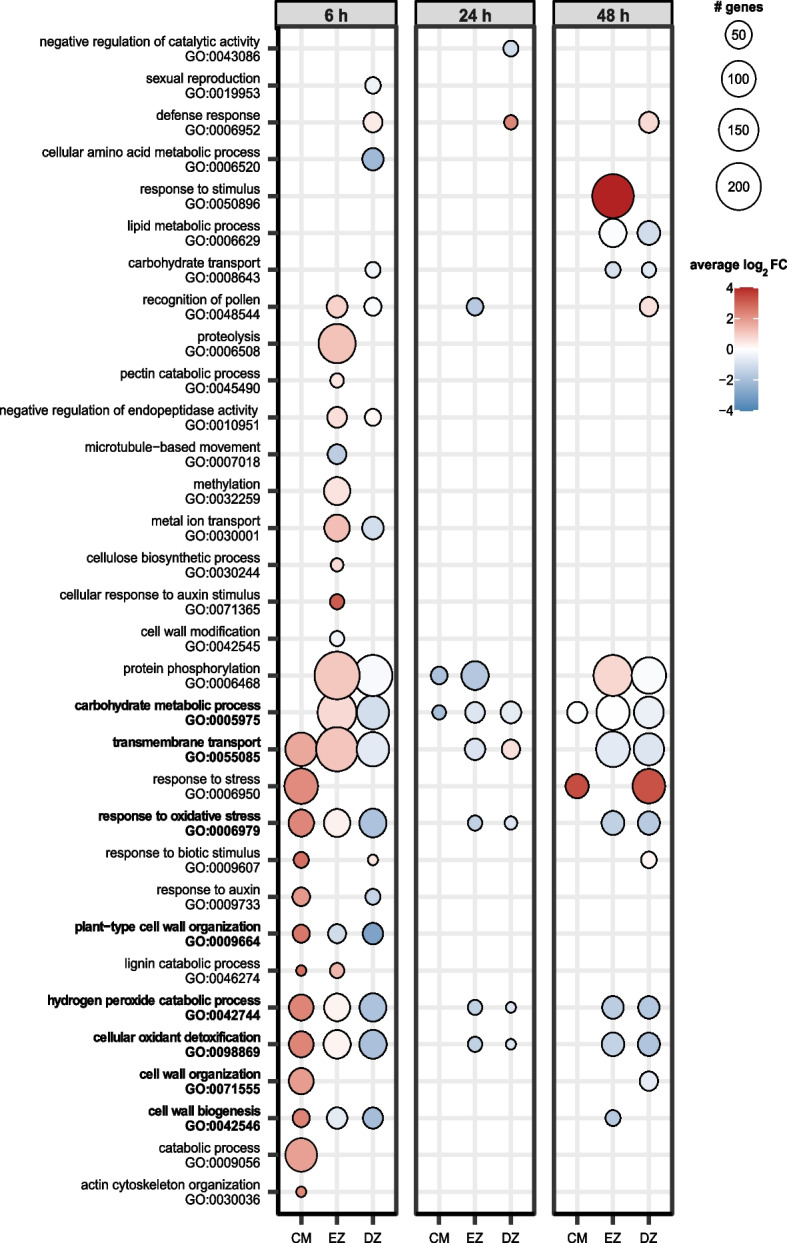
Enriched biological processes based on gene ontology (GO) of differentially expressed genes. Only GO terms with ≥ 10 associated genes are shown. The root zones are root cap and meristem (CM), elongation zone (EZ) and differentiation zone (DZ). The circle size reflects the number of DEGs associated with the respective term and the color indicates the average log_2_FC of these DEGs. Only significantly enriched terms with *p* < 0.05 based on Fisher’s exact test are shown. Bold terms are referred to in the accompanying text

We performed Gene Ontology (GO) enrichment analyses to identify significantly enriched biological processes (Fig. 3) and molecular functions (Figure S3) among the differentially expressed genes in the three analyzed root zones in the time course of water deficit treatment.

After 6 h, all enriched terms related to biological processes were up-regulated in the root cap and meristem, while in the differentiation zone 14 of 17 (82%) of enriched terms were down-regulated. In the elongation zone, 79% (15/19) enriched GO terms were up-regulated, but the direction of regulation within each term varied as seen by the lower average log_2_ fold changes between 0 and 1 Fig. 3). Some stress-responsive GO terms were enriched in all three zones (GO:0006979; GO:0042744; GO:0098869). Another commonly affected term was ‘transmembrane transport’ (GO:0055085), which mainly included aquaporins, ABC transporters, NRT1/PTR family proteins and WAT1-related proteins. Several terms related to cell wall formation and maintenance (GO:0042546; GO:0071555; GO:0009664) were enriched in one or more root zones. These terms mainly encompassed cellulose synthases, expansins and xyloglucan endotransglucosylases. While those genes were strongly up-regulated in the root cap and meristem, they showed strong down-regulation in the differentiation zone, suggesting the maintenance of continued growth through cell wall remodeling and induction of cell division in the meristem.

At 24 h, fewer differentially expressed genes were observed resulting in a lower number of enriched GO terms. The general stress response terms (GO:0006979; GO:0042744; GO:0098869) and the term ‘transmembrane transport’ (GO:0055085) were also enriched but only in the elongation zone and the differentiation zone. The only commonly enriched term in all root zones after 24 h was ‘carbohydrate metabolic process’ (GO:0005975).

After 48 h, ‘carbohydrate metabolic process’ (GO:0005975) was again the only term shared by all root zones, while 80% (8/10) of enriched GO terms of the elongation zone were shared with the differentiation zone. This also encompassed the previously found general stress-related biological processes (GO:0006979; GO:0042744; GO:0098869) and transmembrane transport (GO:0055085).

All enriched molecular functions identified after 6 h of water deficit were up-regulated in the root cap and meristem while 94% (15/16) were down-regulated in the differentiation zone. In the elongation zone 77% (20/26) of enriched molecular functions at that time point were upregulated (Figure S3). Many of the terms were enriched in at least two root zones after 6 h of water deficit. This included some stress-related terms (GO:0004601, GO:0016491, GO:0020037) cell wall-related terms (GO:0016762, GO:0016758) and ‘transmembrane transporter activity’ (GO:0022857). However, a vast number of terms were specific to the elongation zone. Many of these terms were highly specific child terms (GO:0016702, GO:0016614), that derived from the more commonly enriched term ‘oxidoreductase activity’ (GO:0016491). After 24 h, ‘heme binding’ (GO:0020037) a stress-related term was the only one enriched molecular function in all root zones. The few other enriched terms were more root zone-specific. This zone-specificity was also observed in enriched molecular functions after 48 h of water deficit. Still, some stress-related terms (GO:0004601, GO:0016491, GO:0020037) and ‘transmembrane transporter activity’ (GO:0022857) were shared between at least two zones. In summary, these results support the notion, that the response of barley roots to water deficit is root zone and time-dependent, as many different biological processes and molecular functions were enriched across root zones and time points, highlighting the complex and dynamic nature of these responses.

### Weighted gene co-expression analysis identifies modules highly correlated with water deficit

For each of the three root zones, we conducted a weighted gene co-expression network analysis (WGCNA) to identify clusters of highly connected genes (i.e. co-expressed gene modules), associated with water deficit treatment of 6 h, 24 h and 48 h to focus on the treatment effect. To each module, we assigned a specific color name to distinguish between different modules. This provides a complementary approach to the differential expression analysis, by gaining a systems-level understanding of expression patterns.

**Fig. 4:**
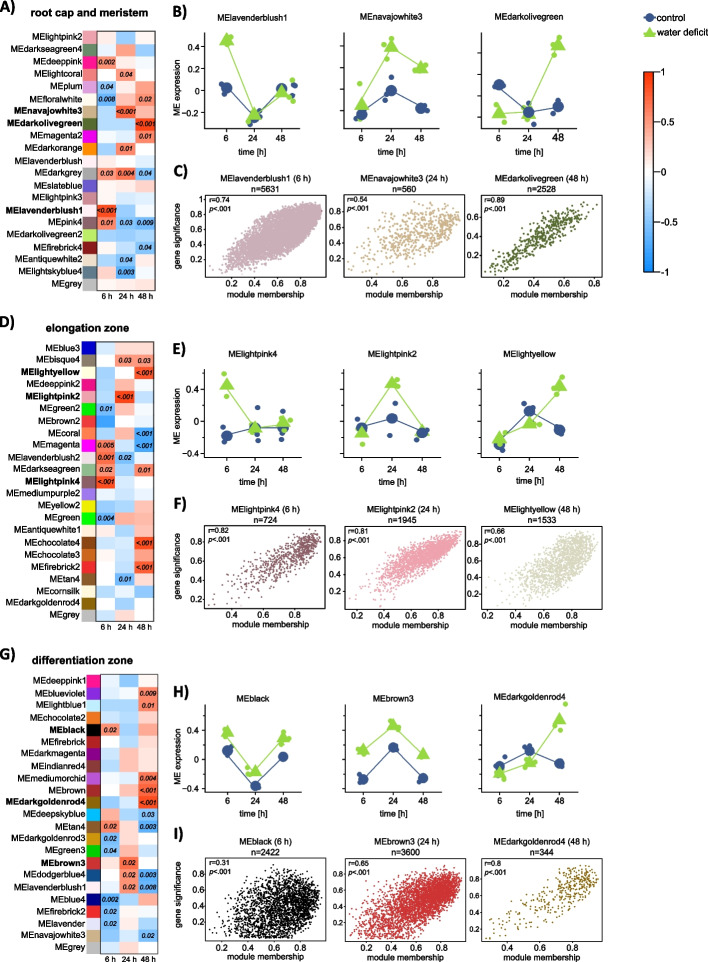
Module-treatment-correlation analysis results. **A**, **D**, **G **Module trait-correlation matrix for co-expression networks derived from the root cap and meristem (**A**), the elongation zone (**D**) and the differentiation zone (**G**). Each column represents a different treatment duration (6 h, 24 h and 48 h of water deficit) and each row represents one co-expression module identified by distinct color names. The color within the matrix cells shows the correlation coefficient between -1 (blue) and 1 (red). Only significant p-values are reported in the cells. One module with the highest significant trait correlation for each time point was chosen for further analyses and marked with bold labels. **B**, **E**, **H** Module eigengene expression pattern over treatment duration in the three selected modules for the root cap and meristem (**B**), the elongation zone (**E**) and the differentiation zone (**H**). Color indicates control (blue) and water deficit samples (green). **C**, **F**, **I **Gene significance versus module membership scatter plots for selected WGCNA modules from root cap and meristem (**C**), elongation zone (**F**) and differentiation zone (**I**). The Pearson correlation coefficient (r) is calculated and reported with the respective p-value

Setting the minimum module size to 30 genes per module, we identified 21, 23 and 23 distinct co-expression modules in the root cap and meristem, the elongation zone and the differentiation zone, respectively (Fig. 4A, D, G). For each module, we calculated its correlation coefficient with the duration of water deficit (Fig. 4A, D, G: color of the matrix cells) and highlighted significant modules (Fig. 4A, D, G: number of p-values in the cells). For downstream analyses, we selected the positively correlated module with the highest significant correlation coefficient for each tissue-by-treatment duration combination (Fig. 4A, D, G: modules highlighted in bold). A comprehensive list of all active genes and their respective module affiliation is provided in Table S3.

Further examination of the module eigengene expression within these selected modules revealed, that in most instances the module eigengene expression was higher in water deficit samples than in control samples at the same time point (Fig. 4B, E, H), indicating that these modules may exhibit a triggered response to water deficit treatment. To understand the relationship between gene expression and the trait water deficit, we assessed the relationship between module membership and gene significance, by calculating the correlation between these two measures for each selected module (see methods). The results showed a strong correlation (r > 0.5) for 8 of 9 modules (Fig. 4C, F, I. These findings suggest that genes with the highest module membership and gene significance in these selected modules are likely associated with water deficit treatment at the respective time points.

### Differential hub gene analysis highlights the root zone specificity of processes and functions under water deficit

**Fig. 5:**
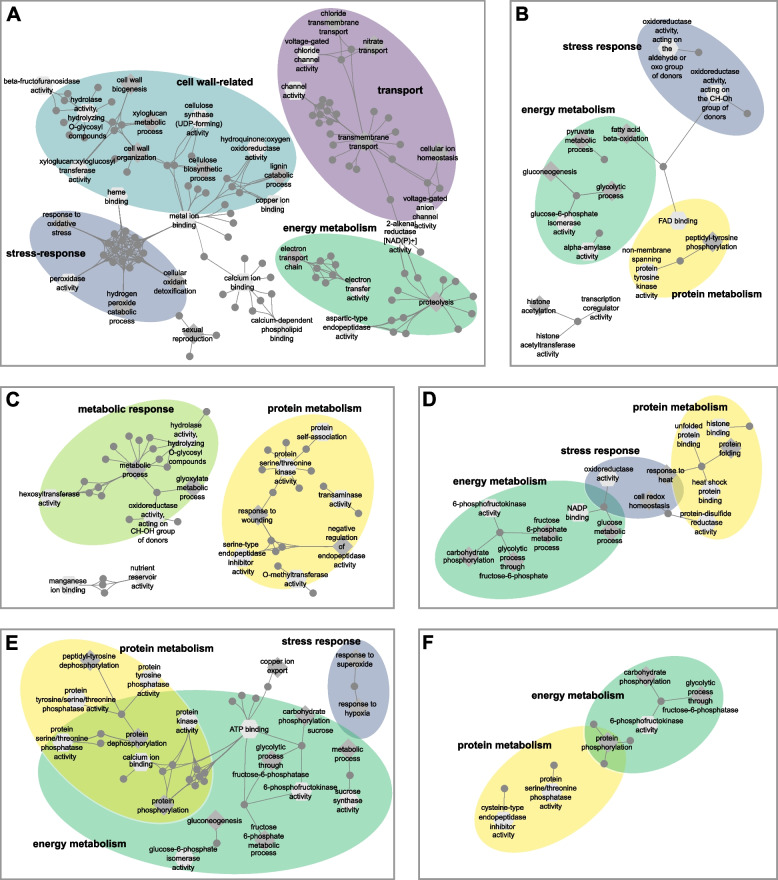
Gene ontology (GO) enrichment analyses of differential hub genes. **A** Root cap and meristem (CM) after 6 h (CM6/MElavenderblush1). **B** CM after 48 h (CM48/MEdarkolivegreen). **C** Elongation zone (EZ) after 6 h (EZ6/MElightpink4). **D** EZ after 24 h (EZ24/MElightpink2). **E** EZ after 48 h (EZ48/MElightyellow). **F** Differentiation zone (DZ) after 48 h (DZ48/darkgoldenrod4). Differential hub genes are represented by grey dots and connected to enriched biological processes (dark grey diamond), and /or enriched molecular functions (light grey hexagon). GO terms associated with similar functions are circled and labeled accordingly

To further investigate the underlying mechanisms that shape water deficit responses, we identified hub genes in each of the modules selected in Fig. 4B, E, H. Hub genes are genes that are highly connected within their co-expression network and are strongly associated with the correlated trait. The identification of hub genes is a key step for reducing the complexity of the analysis and prioritizing the most significant genes. Thus, we set a threshold of ≥ 0.8 for module membership and gene significance, to find all hub genes within the selected modules that showed a high correlation with water deficit treatments. A comprehensive overview of all identified hub genes is listed in Table S4. The number of hub genes varied from very low numbers in all differentiation zone modules (2–28 genes) to up to 418 hub genes in one of the modules of the root cap and meristem. In general, the number of hub genes was lower in 24 h modules than in the 6 h and 48 h modules. We compared the hub genes with the list of previously identified differentially expressed genes. This allowed us to find differentially expressed hub genes that are highly connected, biologically important and consistently associated with water deficit under the same condition. Then we calculated if the observed overlap differed from the expected overlap with Fisher’s exact test or Pearson’s chi-square tests and found a significant overrepresentation of differentially expressed hub genes in six of the nine modules (Figure S4). To identify enriched biological processes and molecular functions covered by the significantly overrepresented differentially expressed hub genes (Figure S4), we performed a functional enrichment analysis of gene ontology (GO) terms for these six comparisons (Fig. 5). In the root cap and meristem modules subjected to 6 h of water deficit (CM6) or 48 h of water deficit (CM48) gene ontology terms mainly fitted into networks associated with energy metabolism, or stress response. While differential hub genes from CM6 also corresponded to cell wall and transport, hub genes from CM48 were additionally associated with stress responses and protein regulation (Fig. 5A, B). In the elongation zone modules subjected to 6 h (EZ6), 24 h (EZ24), or 48 h (EZ48) of water deficit, hub genes were consistently associated with protein metabolism. In EZ6 we additionally identified GO terms related to various metabolic responses. In contrast, EZ24 and EZ48 hub genes were enriched for energy metabolism and stress-related terms (Fig. 5 C-E). Finally, the differential hub genes from the differentiation zone after 48 h of water deficit, were associated with either energy metabolism or protein regulation (Fig. 5F). Overall, the examined responses reflect only a small portion of adaptations occurring during water deficit treatment, since we compared only genes from those modules with the highest Pearson correlation coefficients towards the treatment with the identified differentially expressed genes. Nevertheless, we consider the identified differential hub genes highly relevant, as they showed a strong association with water deficit response based on two different approaches. In summary, these results suggest that although a variety of differential hub genes are involved in water deficit responses, most fit into one of five main categories, with energy metabolism being of particular importance at later time points.

## Discussion

Drought is a significant challenge to agricultural production, which is expected to intensify in occurrence and severity in the foreseeable future due to global warming [45]. Thus, understanding the mechanisms underlying plant responses to water deficit and developing tolerant varieties will improve crop productivity and ensure food safety. Extensive studies on the effects of water deficit on whole roots or root systems have been conducted in major cereals such as rice [46], wheat [47], and barley [48]. However, they do not provide insights into root zone-specific transcriptomic responses to water deficit. To understand the molecular responses to drought stress along the root from the division of root cells via their elongation up to their differentiation is important because they are the underlying causes of whole root system adaptation. In this study, we contribute to closing this knowledge gap by examining the impact of water deficit on barley seminal root development and the transcriptomic response of different seminal root zones of barley.

We observed that root length significantly decreased in barley seedlings after three days of water deficit (Fig. 1A). The detrimental effect of water scarcity on root elongation is consistent with the results of studies in wheat and maize where seminal root lengths were significantly reduced upon water deficit [49, 50]. Moreover, our results showed that root growth rates were already significantly affected on the second day of treatment (Fig. 1B), reflecting an immediate response to changes in water availability, preceding the later observed reduction in root length. Notably, both control and drought-treated plants exhibited similar growth rates after five days. This trend was also observed in barley plants under drought treatment, which showed an equal growth rate at later time points which was comparable to the growth rate of control plants [51]. These findings indicate that the plants adapt dynamically to drought stress over time, which may be influenced by complex feedback signals, triggering the different response phases.

We performed RNA-sequencing of three different root zones of seminal roots after 6 h, 24 h and 48 h of water deficit treatment to further understand the genetic regulation underlying the observed phenotypic changes. Our results showed that the root zone was the main driver for the observed transcriptomic divergence (Fig. 2A). These findings are in accordance with previous studies that have documented that comparable tissue types were the major drivers shaping the transcriptomic landscape in maize primary roots [52] and barley seminal roots [53, 54]. The unique transcriptomic landscape of the different root zones further underlines the relevance of spatial resolution to identify the distinct molecular processes that shape water deficit responses in roots.

We computed pairwise contrast between control and water deficit samples for each time point-by-root zone combination individually to identify genes differentially expressed upon water deficit. We found that the number of differentially expressed genes varied between the three time points (Fig. 2B), indicating a temporal response of barley seedlings to water deficit. In contrast to other studies, the number of responsive genes did not increase over time, which was previously observed after 6 h compared to 24 h in whole roots of barley [25] and maize [50] or after 24 h, 48 h, 96 h and 144 h in pearl millet [55]. Instead, we observed highly dynamic responses after 6 h and 48 h with a higher number of responsive genes compared to a stagnant phase at 24 h, where the number of differentially expressed genes was comparably low in the surveyed root zones. This suggests that roots may adapt to water deficit conditions depending on the duration of exposure. Such a change in gene activity over time was also observed in cotton seedlings subjected to PEG treatment, where the largest number of responsive genes was identified only 3 h after stress induction and the lowest number of responsive genes was detected after 24 h [56].

When comparing the differentially expressed genes in the three root zones across the duration of drought stress, we showed that the majority of genes were unique to the respective root zone-by-stress duration combination but also observed overlapping differentially expressed genes at different treatment durations (Fig. 2C). This is in line with results observed in maize under drought stress, where a large number of differentially expressed genes showed a high time point specificity but also some overlap of drought-responsive genes at more time points [57]. Apart from this temporal response, we also found a spatial response, in which we observed differences in differentially expressed gene numbers between the three root zones (Fig. 2C). Our analysis showed, that the number of responsive genes was always lowest in the root cap and meristem and highest in the elongation zone. This is in line with results from maize seedlings subjected to water stress treatment, where the elongation zone and the cortex were most strongly affected by water deficit [52]. These significant gene expression changes in response to water deficit suggest, that the elongation zone is particularly important for water deficit responses as it might be involved in mediating and maintaining root growth [58] or in adjusting the root structure to cope with water deficit. As observed for the temporal response, some differential genes were shared across root zones, though these were only a minority in comparison to the total number of differentially expressed genes identified in the distinct zones. Such a spatial response was also reported in a comparable experimental setup where the transcriptomes of seminal root zones of barley were subjected to long-term drought stress of 12 days [54]. Regarding the direction of regulation, regulatory directions change between different time points and root zones. This directional change over time was also observed in pearl millet roots under drought stress [55], where it was hypothesized that the regulation of biosynthetic genes especially at later time points may contribute to energy conservation mechanisms since photosynthetic rates under stress are impaired and plants have to prioritize protection over growth to ensure survival [59]. Taken together, these results indicate that water deficit triggers a dynamic and complex spatiotemporal response cascade.

Translocation of various molecules across membranes is crucial for osmotic adjustment under low water conditions [60]. We demonstrated that the gene ontology (GO) terms ‘transmembrane transport’ and ‘transmembrane transporter activity’ were enriched in all root zones at different time points (Fig. 3, Figure S3). Gene models associated with these terms involved aquaporins, ABC transporters and NRT1/PTR family proteins (Table S2).

Aquaporins are major intrinsic proteins that enable the transport of water, gases, metal ions and small neutral solutes across membranes. Alteration of transcript and protein abundance leads to changes in transporter activity, which can have versatile effects under water deficit depending on the aquaporin gene [61]. We found most aquaporins to be up-regulated in the meristematic and elongation zone and a few were down-regulated in the differentiation zone. This aligns with the vital role of aquaporins in root growth, supported by the observation that aquaporins are up-regulated in meristems and growing root tissues in barley [62] and broad beans [63]. Moreover, we observed that aquaporins were upregulated at 6 h and 24 h of stress and only a few were down-regulated after 48 h. This is in line with findings in drought-stressed rice and chickpeas, where aquaporins showed complex regulation under water deficit [64, 65].

Several studies showed that ABC transporters [9, 66] and NRT1/PTR family proteins [67] transport the phytohormone ABA, which in turn regulates aquaporin abundance and activity [68, 69]. In our study, we observed differential expression of these two transporter types, which supports the well-established role of an ABA-dependent signaling pathway during water deficit response [70–72]. High ABA levels induce the transcription of enzymes which are important for maintaining the cell redox homeostasis by scavenging reactive oxygen species (ROS) such as oxidases, reductases and peroxidases [73, 74]. We found some stress-responsive GO terms, entailing various of these enzymes (Fig. 3, Figure S3). Among them, peroxidases were the most prominent differentially-regulated genes. The non-uniform differential regulation of genes involved in the redox system was also detected in drought-stressed maize [57] and suggests a complex mechanism for regulating cell redox homeostasis, cell wall integrity and cell growth under water deficit conditions. In summary, our analysis of differential gene expression proposes that water deficit triggers a dynamic and sophisticated spatiotemporal response system involving the alteration of various functions and pathways.

To complement our differential expression analysis, we employed a weighted gene co-expression analysis to identify co-expressed gene modules associated with water deficit treatment. We conducted the analyses separately for each root zone, to focus on the treatment effect. For each root zone-by-duration of drought stress combination, we selected the module with the strongest positive correlation toward treatment for further downstream analysis. Comparisons of eigengene expression in the selected modules revealed a treatment-specific pattern. We then identified hub genes in each selected module, which are the regulatory key genes [18] and identified a significant overrepresentation of differentially expressed hub genes in six of the nine modules (Figure S4). GO enrichment analysis of differential hub genes in these six modules revealed unique and conserved GO term categories in the modules identified in different root zone-by-drought treatment combinations. Most GO term categories were present in most root zone-by-treatment combinations. For instance, the categories “energy metabolism” modulating carbohydrate and sugar metabolic processes (Fig. 5A, C-F) or “protein metabolism” (Fig. 5B-F) were present in five of six combinations and “stress response” in four of six combinations (Fig. 5A, B, D, E) highlighting the importance of GO terms within these categories in drought stress response. Importantly, the individual GO terms and subsequently the hub genes in these conserved categories were different, suggesting a wide range of functional adjustments triggered by water deficit. In contrast to these conserved categories “cell wall-related” functions and “transport” were uniquely observed in the root cap and the meristem at 6 h (Fig. 5A). Cell walls are dynamic interfaces with the environment and undergo remarkable adaptive alterations in their composition and structure under stress [75]. Up-regulation of cell wall-related genes was also observed in maize [52] and wheat [47] roots under drought stress. The balance between ROS and peroxidases plays a crucial role in cell wall loosening and growth maintenance. The prominence of peroxidases as a group of differentially expressed hub genes exclusively in the root cap and meristem at 6 h (Fig. 5A) might highlight their pivotal role in the initial response to water deficit in the early stages [47, 57]. The enrichment of GO terms associated with transport solely among differential hub genes in the root cap and meristem at 6 h suggests that up-regulation of transporters might be associated with the relocation of essential substrates to maintain cellular homeostasis [47] and enable the transmission of signal molecules necessary to trigger the initial water stress-responsive pathways [76]. Taken together, the integration of differential expression and co-expression network analysis provides a comprehensive overview of the processes and functions underlying plant responses to water deficit stress.

## Conclusion

Our research highlights the spatiotemporal response cascades in barley seedling root zones triggered by water deficit for two days. The observed root zone and time-specific mechanisms further underline the importance of investigating stress response mechanisms in a zone-specific manner instead of whole root systems, while considering the temporal dynamics. Our findings contribute to a better understanding of distinct and dynamical changes that shape the plant responses to water deficit and might provide additional targets to enhance plant resilience and reduce the negative impacts of water deficit in agriculture.

### Supplementary Information

**Additional file 1: Figure S1.** Library sizes of RNA-sequencing samples derived from three different root zones and time points. The root zones are root cap and meristem (CM), elongation zone (EZ) and differentiation zone (DZ). Blue bars represent control sample libraries, red bars represent water deficit sample libraries. The shade reflects the time point (6 h, 24 h or 48 h) with darker shades for later time points.

**Additional file 2: Figure S2.** Volcano plots of differentially expressed genes. Significantly up-regulated (FDR < 5%, log_2_FC > 1) differentially expressed genes (DEGs) are shown in yellow, down-regulated (FDR < 5%, log_2_FC < -1) DEGs are shown in purple. The total number of DEGs are shown in the upper left and right corners of each panel. DEGs that do not exceed the significance threshold are depicted in grey. DEGs were calculated between control and water deficit samples for each root zone and time point (6 h, 24 h and 48 h) separately. The root zones are root cap and meristem (CM), elongation zone (EZ) and differentiation zone (DZ).

**Additional file 3: Figure S3.** Enriched molecular functions based on gene ontology (GO) of differentially expressed genes. Only GO terms with ≥ 10 associated genes are shown. The root zones are root cap and meristem (CM), elongation zone (EZ) and differentiation zone (DZ). The circle size reflects the number of DEGs associated with the respective term and color indicates the average log_2_FC of these DEGs. Only significantly enriched terms with *p* < 0.05 based on a Fisher’s exact test are shown.

**Additional file 4: Figure S4.** Venn diagrams with a significant overrepresentation of differential hub genes. Comparison of hub genes (yellow circle) and differentially expressed genes (blue circle) from the corresponding root zone-time point combinations. The root zones are root cap and meristem (CM), elongation zone (EZ) and differentiation zone (DZ). DEGs were identified after 6 h, 24 h and 48 h. Deviations between expected and observed overlap were calculated based on either Fisher’s exact test (*n* < 5) or Pearson’s chi-square test (*n* ≥ 5) with *p* < 0.05.

**Additional file 5: Table S1.** Overview of RNA-sequencing raw read output and consecutive pseudo alignment results.

**Additional file 6: Table S2.** List of significantly differentially expressed genes between control and water deficit samples after 6 h, 24 h and 48 h of treatment in three distinct root zones.

**Additional file 7: Table S3.** List of all active genes and their assigned modules including gene significance (GS) for the water deficit treatments at 6 h, 24 h and 48 h (D6, D24 and D48) and their functional description.

**Additional file 8: Table S4.** List of all identified hub genes within selected modules that show a high correlation with water deficit at one respective time point including gene descriptions.

## Data Availability

The dataset generated and analyzed during the current study is available in the NCBI SRA repository under BioProject accession number PRJNA988922 (https://www.ncbi.nlm.nih.gov/sra/PRJNA988922). All data generated or analyzed during this study are included in this published article and its supplementary information files.

## Abbreviations

ABA: Abscisic acid
CM: Root cap and meristem
DREB: Dehydration-responsive element binding
DZ: Differentiation zone
EMM: Estimated marginal means
EZ: Elongation zone
GO: Gene ontology
MDS plot: Multidimensional scaling plot
PEG8000: Polyethylene glycol
PFP: Pyrophosphate‐fructose 6‐phosphate 1‐phosphotransferase
ROS: Reactive oxygen species
WGCNA: Weighted gene co-expression network analysis
