# Supplementary material for: Spatiotemporal transcriptomic plasticity in barley roots: unravelling water deficit responses in distinct root zones
Source: BMC Genomics. 2024 Jan 19;25:79. doi: 10.1186/s12864-024-10002-0 (PMC10799489; doi:10.1186/s12864-024-10002-0)
Supplement: Supplementary file 1 — Additional file 1: Figure S1. Library sizes of RNA-sequencing samples derived from three different root zones and time points. The root zones are root cap and meristem (CM), elongation zone (EZ) and differentiation zone (DZ). Blue bars represent control sample libraries, red bars represent water deficit sample libraries. The shade reflects the time point (6 h, 24 h or 48 h) with darker shades for later time points. [file 12864_2024_10002_MOESM1_ESM.pdf]

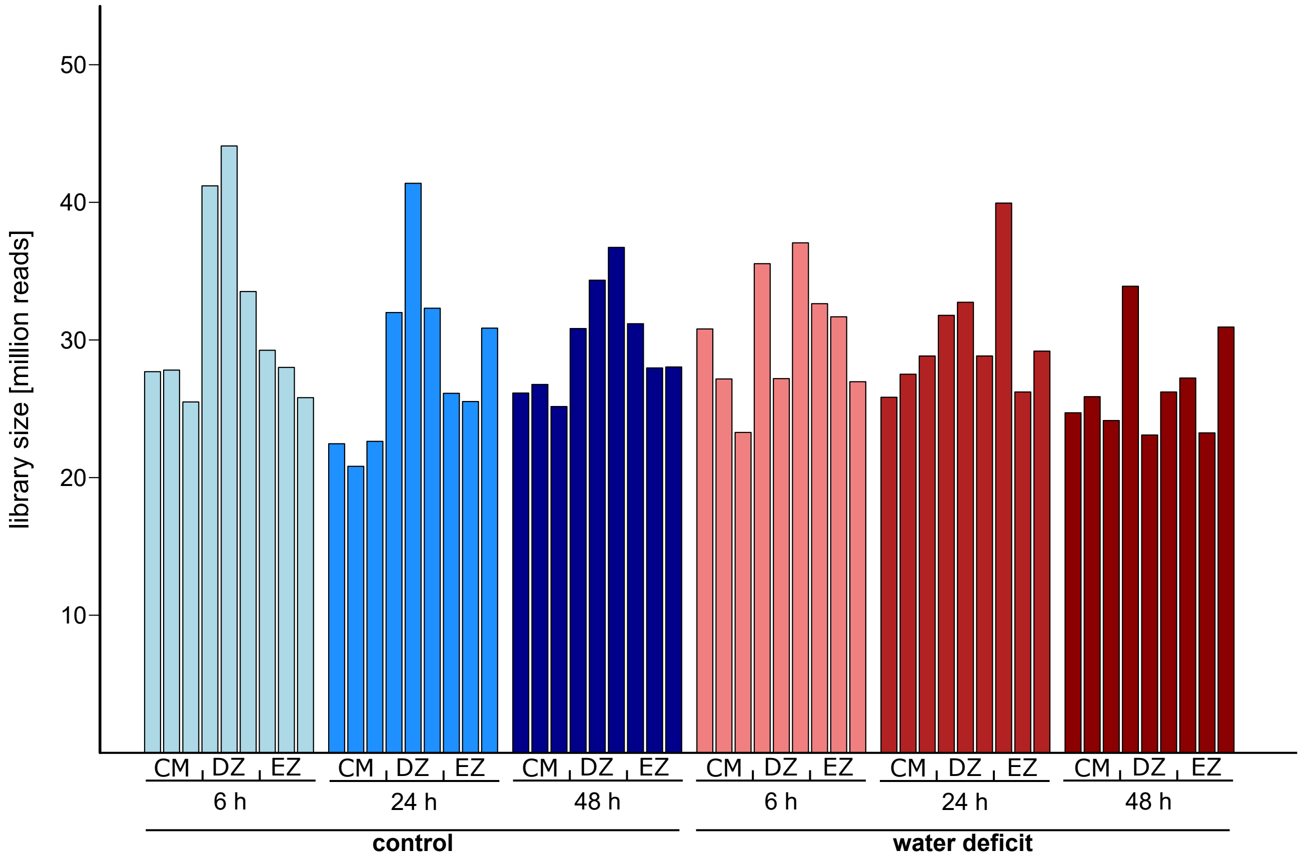

Fig. S1: Library sizes of RNA-sequencing control and water deficit samples derived from three different root zones and time points. The root zones are root cap and meristem (CM), elongation zone (EZ) and differentiation zone (DZ). Blue bars represent control sample libraries, red bars represent water deficit sample libraries. The shade reflects the time point (6 h, 24 h or 48 h) with darker shades for later time points.
