# Supplementary material for: Spatiotemporal transcriptomic plasticity in barley roots: unravelling water deficit responses in distinct root zones
Source: BMC Genomics. 2024 Jan 19;25:79. doi: 10.1186/s12864-024-10002-0 (PMC10799489; doi:10.1186/s12864-024-10002-0)
Supplement: Supplementary file 2 — Additional file 2: Figure S2. Volcano plots of differentially expressed genes. Significantly up-regulated (FDR < 5%, log2FC > 1) differentially expressed genes (DEGs) are shown in yellow, down-regulated (FDR < 5%, log2FC < -1) DEGs are shown in purple. The total number of DEGs are shown in the upper left and right corners of each panel. DEGs that do not exceed the significance threshold are depicted in grey. DEGs were calculated between control and water deficit samples for each root zone and time point (6 h, 24 h and 48 h) separately. The root zones are root cap and meristem (CM), elongation zone (EZ) and differentiation zone (DZ). [file 12864_2024_10002_MOESM2_ESM.pdf]

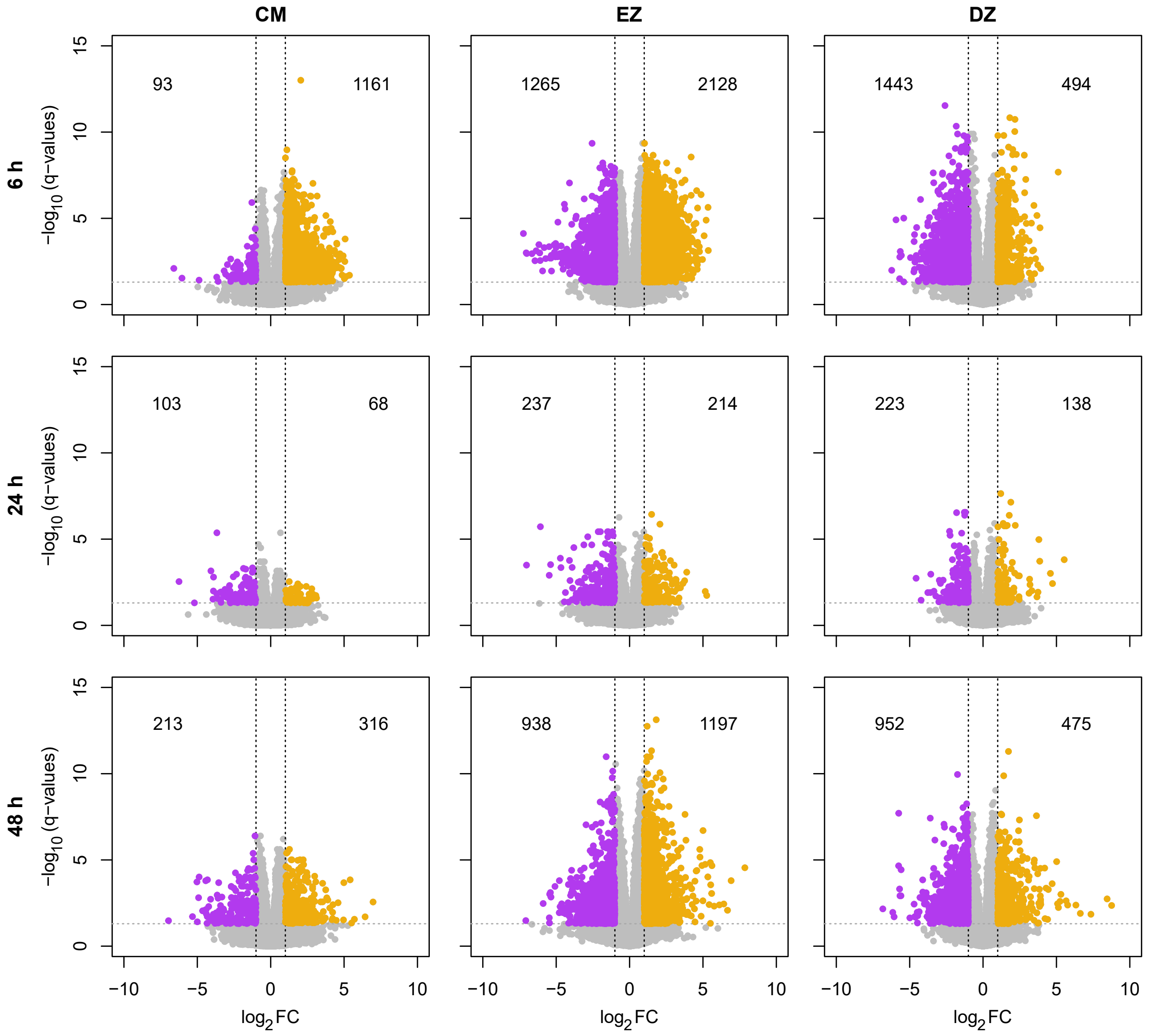

Fig. S2: Volcano plots of differentially expressed genes. Significantly (FDR <5%, |log2FC| >1) up-regulated differentially expressed genes (DEGs) are shown in yellow, down-regulated DEG are shown in purple. The total number of DEGs are shown in the upper left and right corners of each panel. DEGs that do not exceed the significance threshold are depicted in grey. DEGs were calculated between control and water deficit samples for each root zone and time point (6 h, 24 h and 48 h) separately. The root zones are root cap and meristem (CM), elongation zone (EZ) and differentiation zone (DZ).
