# Supplementary material for: Spatiotemporal transcriptomic plasticity in barley roots: unravelling water deficit responses in distinct root zones
Source: BMC Genomics. 2024 Jan 19;25:79. doi: 10.1186/s12864-024-10002-0 (PMC10799489; doi:10.1186/s12864-024-10002-0)
Supplement: Supplementary file 3 — Additional file 3: Figure S3. Enriched molecular functions based on gene ontology (GO) of differentially expressed genes. Only GO terms with ≥ 10 associated genes are shown. The root zones are root cap and meristem (CM), elongation zone (EZ) and differentiation zone (DZ). The circle size reflects the number of DEGs associated with the respective term and color indicates the average log2FC of these DEGs. Only significantly enriched terms with p < 0.05 based on a Fisher’s exact test are shown. [file 12864_2024_10002_MOESM3_ESM.pdf]

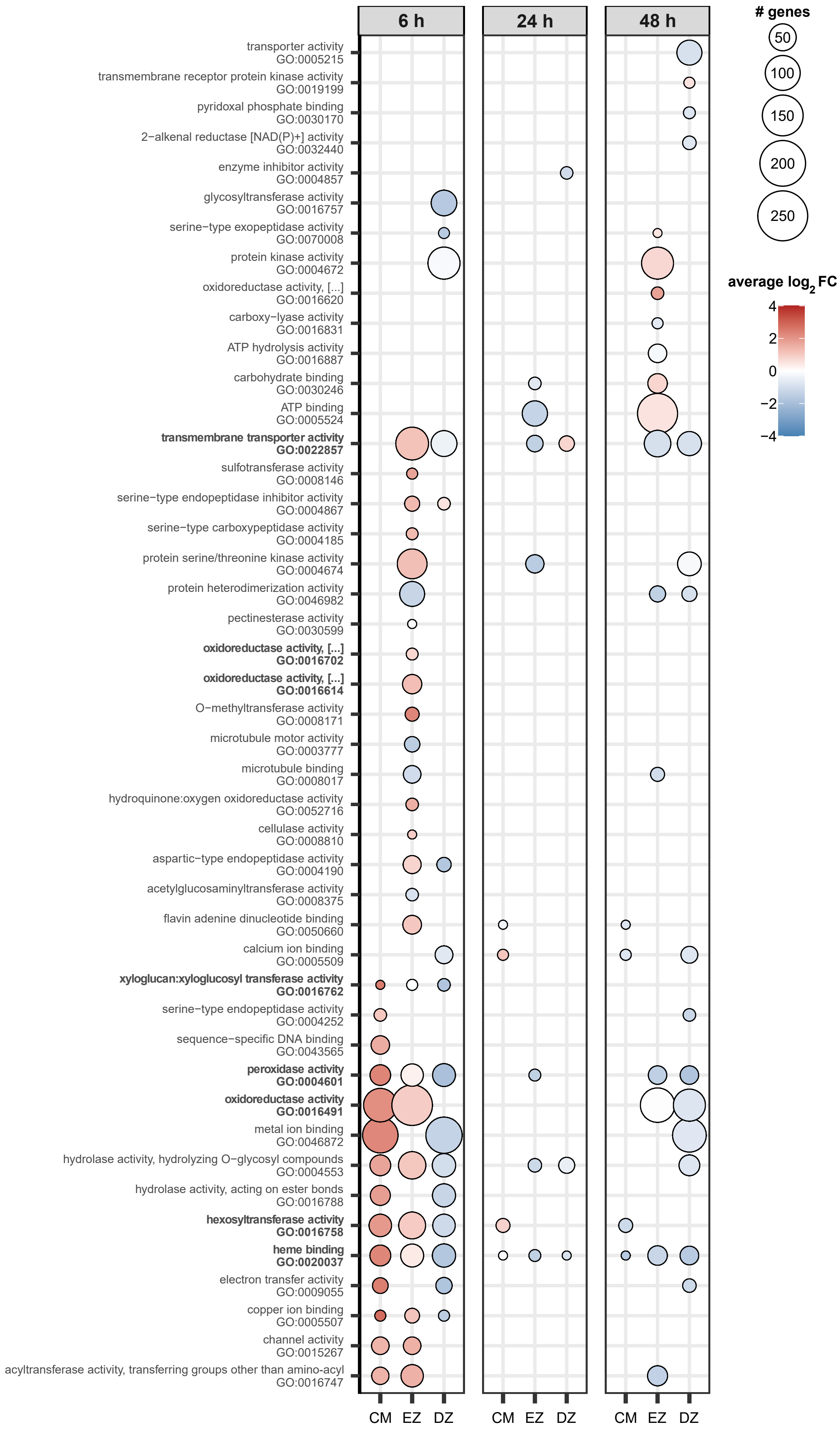

Fig. S3: Enriched molecular functions based on gene ontology (GO) enrichment analyses of differentially expressed genes (DEGs) responsive to water deficit treatment. Only GO terms with ≥10 associated genes are shown. The root zones are root cap and meristem (CM), elongation zone (EZ) and differentiation zone (DZ). The circle size reflects the number of DEGs associated with the respective term and color indicates the average log2FC of these DEGs. Only significantly enriched terms with $p < 0.05$ based on a Fisher's exact test are shown.
