# Supplementary material for: Spatiotemporal transcriptomic plasticity in barley roots: unravelling water deficit responses in distinct root zones
Source: BMC Genomics. 2024 Jan 19;25:79. doi: 10.1186/s12864-024-10002-0 (PMC10799489; doi:10.1186/s12864-024-10002-0)
Supplement: Supplementary file 4 — Additional file 4: Figure S4. Venn diagrams with a significant overrepresentation of differential hub genes. Comparison of hub genes (yellow circle) and differentially expressed genes (blue circle) from the corresponding root zone-time point combinations. The root zones are root cap and meristem (CM), elongation zone (EZ) and differentiation zone (DZ). DEGs were identified after 6 h, 24 h and 48 h. Deviations between expected and observed overlap were calculated based on either Fisher’s exact test (n < 5) or Pearson’s chi-square test (n ≥ 5) with p < 0.05. [file 12864_2024_10002_MOESM4_ESM.pdf]

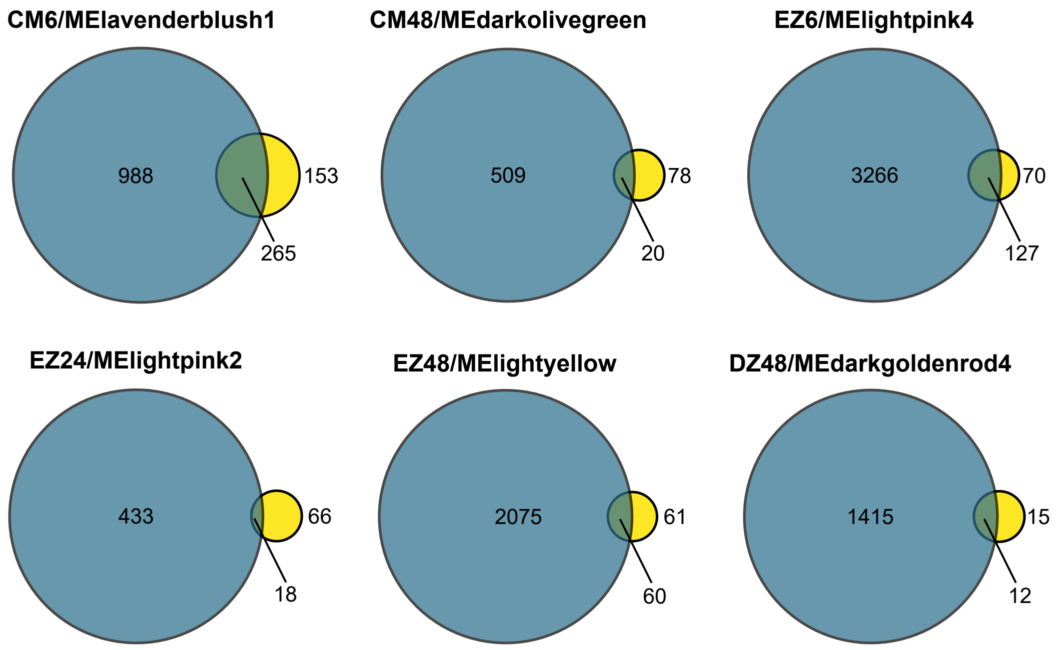

Fig. S4: Venn diagrams with a significant overrepresentation of differential hub genes among the observed overlap between hub genes (yellow) and differentially expressed genes (DEGs, blue) from the corresponding root-zone-time-point analysis. The root zones are root cap and meristem (CM), elongation zone (EZ) and differentiation zone (DZ). DEGs were identified after 6 h, 24 h and 48 h. Deviations between expected and observed overlap were calculated based on either Fisher's exact test ($n < 5$) or Pearson's chi-square test ($n \geq 5$) with $p < 0.05$.
