# Supplementary material for: Spatiotemporal transcriptomic plasticity in barley roots: unravelling water deficit responses in distinct root zones
Source: BMC Genomics. 2024 Jan 19;25:79. doi: 10.1186/s12864-024-10002-0 (PMC10799489; doi:10.1186/s12864-024-10002-0)
Supplement: Supplementary file 5 — Additional file 5: Table S1. Overview of RNA-sequencing raw read output and consecutive pseudo alignment results. [file 12864_2024_10002_MOESM5_ESM.docx]

**Table S1:** Overview of RNA-sequencing raw read output and consecutive pseudo alignment results.

| **sample** | **time point** | **root zone^1^** | **replicate** | **total - raw** | **total - after quality trimming** | **total - pseudo aligned** | **pseudo alignment rate on transcriptome [%]** |
| --- | --- | --- | --- | --- | --- | --- | --- |
| control | 6 h | CM | 1 | 38,271,433 | 37,163,856 | 32,841,636 | 88.4 |
|  |  | CM | 2 | 37,898,712 | 36,728,501 | 32,465,389 | 88.4 |
|  |  | CM | 3 | 34,907,334 | 33,985,123 | 30,077,560 | 88.5 |
| control | 24 h | CM | 1 | 45,845,418 | 44,373,838 | 38,761,395 | 87.4 |
|  |  | CM | 2 | 41,267,338 | 40,119,651 | 35,083,869 | 87.4 |
|  |  | CM | 3 | 49,371,893 | 47,878,113 | 41,672,825 | 87.0 |
| control | 48 h | CM | 1 | 37,072,122 | 35,964,237 | 31,513,125 | 87.6 |
|  |  | CM | 2 | 38,270,661 | 36,880,113 | 32,543,651 | 88.2 |
|  |  | CM | 3 | 35,220,661 | 34,174,479 | 30,257,729 | 88.5 |
| water deficit | 6 h | CM | 1 | 42,932,448 | 41,487,410 | 36,446,539 | 87.8 |
|  |  | CM | 2 | 37,874,233 | 36,547,198 | 32,079,574 | 87.8 |
|  |  | CM | 3 | 32,596,295 | 31,524,285 | 27,503,289 | 87.2 |
| water deficit | 24 h | CM | 1 | 30,689,031 | 29,806,782 | 26,151,913 | 87.7 |
|  |  | CM | 2 | 33,672,426 | 32,737,765 | 28,842,195 | 88.1 |
|  |  | CM | 3 | 32,090,683 | 31,074,814 | 27,171,978 | 87.4 |
| water deficit | 48 h | CM | 1 | 33,838,342 | 32,882,276 | 29,000,108 | 88.2 |
|  |  | CM | 2 | 35,189,529 | 34,255,444 | 30,340,358 | 88.6 |
|  |  | CM | 3 | 33,275,519 | 32,323,703 | 28,336,664 | 87.7 |
| control | 6 h | EZ | 1 | 33,275,877 | 31,984,443 | 28,894,358 | 90.3 |
|  |  | EZ | 2 | 38,068,089 | 36,720,916 | 32,286,535 | 87.9 |
|  |  | EZ | 3 | 36,501,929 | 35,457,759 | 31,025,494 | 87.5 |
| control | 24 h | EZ | 1 | 48,450,221 | 46,738,095 | 41,181,859 | 88.1 |
|  |  | EZ | 2 | 42,746,342 | 41,408,019 | 36,008,218 | 87.0 |
|  |  | EZ | 3 | 39,836,075 | 38,508,770 | 34,055,354 | 88.4 |
| control | 48 h | EZ | 1 | 40,726,853 | 39,364,195 | 34,125,205 | 86.7 |
|  |  | EZ | 2 | 37,212,735 | 35,950,622 | 30,946,295 | 86.1 |
|  |  | EZ | 3 | 36,293,885 | 35,110,348 | 30,647,077 | 87.3 |
| water deficit | 6 h | EZ | 1 | 41,278,973 | 40,042,854 | 35,469,171 | 88.6 |
|  |  | EZ | 2 | 40,192,129 | 39,139,500 | 34,455,089 | 88.0 |
|  |  | EZ | 3 | 34,763,731 | 33,708,607 | 29,282,607 | 86.9 |
| water deficit | 24 h | EZ | 1 | 29,294,253 | 28,414,685 | 24,355,731 | 85.7 |
|  |  | EZ | 2 | 37,418,864 | 36,446,301 | 31,665,116 | 86.9 |
|  |  | EZ | 3 | 32,048,785 | 31,156,152 | 26,665,332 | 85.6 |
| water deficit | 48 h | EZ | 1 | 34,298,728 | 33,304,255 | 29,506,710 | 88.6 |
|  |  | EZ | 2 | 29,414,056 | 28,462,759 | 25,143,561 | 88.3 |
|  |  | EZ | 3 | 38,914,805 | 37,841,922 | 33,565,400 | 88.7 |
| control | 6 h | DZ | 1 | 52,335,006 | 50,856,898 | 44,631,985 | 87.8 |
|  |  | DZ | 2 | 56,297,907 | 54,503,122 | 47,602,516 | 87.3 |
|  |  | DZ | 3 | 42,736,536 | 41,391,519 | 36,135,719 | 87.3 |
| control | 24 h | DZ | 1 | 44,996,093 | 43,651,274 | 38,700,707 | 88.7 |
|  |  | DZ | 2 | 39,792,327 | 38,757,327 | 34,126,031 | 88.1 |
|  |  | DZ | 3 | 38,351,949 | 37,121,165 | 32,418,175 | 87.3 |
| control | 48 h | DZ | 1 | 40,341,175 | 39,119,596 | 33,444,779 | 85.5 |
|  |  | DZ | 2 | 44,144,996 | 42,763,789 | 37,302,682 | 87.2 |
|  |  | DZ | 3 | 46,695,821 | 45,294,801 | 39,630,812 | 87.5 |
| water deficit | 6 h | DZ | 1 | 45,654,602 | 44,261,690 | 38,265,693 | 86.5 |
|  |  | DZ | 2 | 35,027,790 | 33,987,585 | 29,250,908 | 86.1 |
|  |  | DZ | 3 | 47,278,180 | 45,710,822 | 39,816,696 | 87.1 |
| water deficit | 24 h | DZ | 1 | 42,770,651 | 41,526,560 | 34,734,978 | 83.6 |
|  |  | DZ | 2 | 39,336,347 | 38,232,417 | 32,390,090 | 84.7 |
|  |  | DZ | 3 | 29,343,570 | 28,540,654 | 24,249,198 | 85.0 |
| water deficit | 48 h | DZ | 1 | 42,851,421 | 41,674,128 | 36,559,801 | 87.7 |
|  |  | DZ | 2 | 30,380,301 | 29,498,209 | 25,107,025 | 85.1 |
|  |  | DZ | 3 | 33,937,559 | 32,862,075 | 28,286,058 | 86.1 |
| **Average** | | | | 38,764,679 | 37,581,878 | 32,833,755 | 87.4 |
| **Minimum** | | | | 29,294,253 | 28,414,685 | 24,249,198 | 83.6 |
| **Maximum** | | | | 56,297,907 | 54,503,122 | 47,602,516 | 90.3 |

**^1^** CM: root cap and meristem, EZ: elongation zone, DZ: differentiation zone
